# Development and validation of a novel triplex droplet digital PCR assay for simultaneous detection of African swine fever virus, pseudorabies virus, and porcine parvovirus

**DOI:** 10.3389/fmicb.2025.1710807

**Published:** 2026-01-06

**Authors:** Jiarong Yu, Lu Zhu, Yuanyuan Zuo, Shengbin Gao, Qinghua Wang, Jiao Xu, Linlin Fang, Yumeng Liu, Shuang Liu, Xiaozhen Wang, Xiaohua Wang, Yingli Wang, Jingming Li, Jingyue Bao, Zhiliang Wang

**Affiliations:** 1 China Animal Health and Epidemiology Center, Qingdao, China; 2 College of Veterinary Medicine, Qingdao Agricultural University, Qingdao, China

**Keywords:** clinical diagnosis, multiplex droplet digital PCR, ASFV, PRV, PPV

## Abstract

Coinfection with multiple viruses significantly complicates swine disease management. DNA viruses including African swine fever virus (ASFV), pseudorabies virus (PRV), and porcine parvovirus (PPV) are causing reproductive failure in sows, with overlapping clinical presentations that challenge differential diagnosis. To address this, we developed a novel triplex droplet digital PCR (ddPCR) assay using three distinct fluorescent probe sets for simultaneous detection of these three viruses. The multiplex ddPCR demonstrated superior analytical performance with high specificity, sensitivity, and reproducibility. When compared to conventional quantitative PCR (qPCR), the ddPCR assay achieved limits of detection (LOD) of 0.08, 3.41, and 3.38 copies/μL for ASFV, PRV, and PPV respectively-representing approximately 10-fold lower LOD values than corresponding qPCR methods. Cross-reactivity tests confirmed absolute specificity against other important swine pathogens. Clinical validation using 217 field samples revealed marked diagnostic advantages: ddPCR detected positive cases in 137 samples (63.1%) versus 115 (53.0%) by qPCR. Species-specific positivity rates showed ddPCR improved detection by 5.99% (ASFV), 0.46% (PRV), and 3.69% (PPV) compared to qPCR. Concordance analysis demonstrated strong agreement between methods (94.01–99.54%), with ddPCR showing superior sensitivity particularly for low-viral-load samples. This study establishes the first multiplex digital PCR platform for simultaneous differential diagnosis of ASFV, PRV, and PPV. The assay provides a robust tool for enhanced surveillance and control of these economically significant pathogens in swine populations.

## Introduction

The simultaneous presence of multiple pathogens in large-scale pig farms, including African swine fever virus (ASFV), pseudorabies virus (PRV), and porcine parvovirus (PPV), poses significant challenges to swine health management. These pathogens can cause of reproductive disorders in sows, negatively impacting breeding herd productivity and creating unprecedented obstacles for disease control and sustainable pig production. African swine fever (ASF), caused by ASFV, is an acute, highly lethal, and transmissible disease listed as a notifiable disease by the World Organisation for Animal Health (WOAH). This large, complex DNA virus infects both domestic pigs and wild boars, manifesting clinically with hyperthermia, cutaneous cyanosis, and severe lymphoid tissue necrosis. The disease is characterized by near-100% morbidity and mortality rates (Dixon et al., 2019). Since its emergence in China in 2018, ASF has caused devastating economic losses to the swine industry (Huang et al., 2021). You et al. (2021) developed a systematic valuation framework to evaluate the economic losses caused by the African Swine Fever (ASF) outbreak in China from August 2018 to July 2019. Their analysis revealed that the epidemic impacted nearly all economic sectors, with losses equivalent to 0.78% of China’s gross domestic product. Pseudorabies virus (PRV), also known as 1 porcine herpesvirus, is an enveloped α-herpesvirus containing a double-stranded DNA genome (Romero et al., 1997). Upon infection, PRV demonstrates rapid intracellular replication, leading to systemic lesions and neurological dysfunction. Clinical presentations include pyrexia, emesis, and diarrhea, with significant implications for piglet mortality and herd health (Weidner-Glunde et al., 2020). In addition, PRV can infect a wide range of animals, including pigs, dogs, cats, cattle, sheep, goats, captive minks, wild foxes, captive foxes, wolves, and lynx. The cross-species transmission of PRV from pigs to humans has also garnered growing attention (Liu et al., 2022). Porcine parvovirus (PPV), a non-enveloped, single-stranded negative-sense DNA virus, is a well-established cause of reproductive failure in sows and respiratory distress in growing pigs. The virus exhibits remarkable environmental stability, contributing to its ubiquitous presence in swine populations (Allan et al., 1999; Cadar et al., 2011). Between 2020 and 2023, Chen et al. (2025) performed an epidemiological investigation on 1,534 samples in China’s southwest region. Their findings revealed a 3% abortion rate attributed to PPV2 infection, alongside antibody positivity rates ranging from 73.03 to 90%. These results underscore the persistent and widespread prevalence of PPV in the surveyed population. The overlapping clinical presentations of these three viral diseases complicate field diagnosis. Furthermore, the documented occurrence of mixed or secondary infections among ASFV, PRV, and PPV in swine populations underscores the critical need for rapid, accurate laboratory diagnostic methodologies (Liu et al., 2023; Pérez et al., 2012).

Current diagnostic approaches for clinical sample detection prioritize convenience, sensitivity, and rapidity. Nucleic acid amplification techniques (NAATs) such as polymerase chain reaction (PCR), reverse transcription PCR (RT-PCR), and real-time quantitative PCR (qPCR/qRT-PCR) remain the gold standard methods (Espy et al., 2006; Zhu et al., 2020). While conventional PCR and RT-PCR provide qualitative results through gel electrophoresis, they lack quantitative precision and require post-amplification processing. Quantitative PCR platforms offer improved speed and sensitivity compared to end-point analysis, but their quantitative accuracy depends on standard curve calibration and cycle threshold (Ct) value interpretation, which may introduce variability between assays (Sluijter et al., 2006). Alternative methodologies have emerged to address these limitations. Isothermal amplification techniques like loop-mediated isothermal amplification (LAMP) and CRISPR-based detection systems coupled with flow Immunoassay promising sensitivity and rapid amplification capabilities. However, these methods face challenges with non-specific amplification leading to false-positive results (Kim et al., 2023). Additionally, both LAMP and CRISPR-based approaches impose stringent requirements on target sequence selection: LAMP necessitates relatively long target sequences with specific structural motifs, while CRISPR systems require precise protospacer adjacent motifs (PAMs) for guide RNA binding. These technical constraints limit their universal applicability for routine diagnostic use (Ding et al., 2019).

Droplet digital PCR (ddPCR) represents a third-generation nucleic acid amplification technology that enables absolute quantification of nucleic acid targets through Poisson statistics, eliminating reliance on standard curves or reference materials (Li et al., 2018; Pinheiro et al., 2012). Compared to conventional PCR and quantitative PCR (qPCR), this technique demonstrates superior sensitivity, specificity, accuracy, and reproducibility (Huggett and Whale, 2013). A particular strength lies in its multiplexing capability, where multiple targets can be simultaneously detected across distinct fluorescent channels while maintaining exceptional analytical performance (Gaňová et al., 2021). Recent applications highlight its diagnostic versatility: Zhu et al. (2022) developed a multiplex ddPCR system capable of differentiating enterovirus (EV), parechovirus (HPeV), and herpes simplex viruses 1/2 (HSV1/2) in clinical specimens. In oncology research, de Kock et al. (2021) employed multiplex ddPCR to simultaneously detect EGFR sensitizing mutations and resistance-associated variants with single-molecule resolution (de Kock et al., 2021). The technology also shows genotype discrimination capabilities, as demonstrated by Ren’s et al. (2018) dual-probe ddPCR assay distinguishing wild-type pseudorabies virus (PRV) from vaccine strains.

Collectively, these applications underscore ddPCR’s potential to revolutionize clinical diagnostics through its unprecedented precision, multiplexing capacity, and absolute quantification capabilities. As technical advancements continue to enhance throughput and reduce costs, multiplex ddPCR is poised to become a cornerstone of next-generation molecular diagnostics. Here in we present a novel triplex droplet digital PCR (ddPCR) assay for the simultaneous differential detection of ASFV, PRV, and PPV—a diagnostic approach that has not been previously reported. This study represents the first demonstration of a multiplex ddPCR platform capable of distinguishing these three economically significant swine pathogens in a single reaction. Through rigorous validation using clinical specimens, we have confirmed the assay’s exceptional sensitivity in detecting low-viral-load samples and its unique capability to identify mixed infections with high diagnostic specificity. The method’s rapid turnaround time (≤2.5 h) and absolute quantification without reliance on standard curves offer significant advantages over conventional PCR-based methods. These findings providing a critical tool for early outbreak detection, surveillance, and informed decision-making in regional disease control programs. The developed triplex assay demonstrates remarkable potential to enhance biosecurity measures by enabling rapid differential diagnosis of co-circulating swine pathogens, thus offering a scientific foundation for evidence-based containment strategies.

## Materials and methods

### Genomic DNA/RNA samples and clinical tissue samples

The 217 clinical samples from different pig herds, including lymph nodes, spleen and blood of each dead pig, were collected in Shandong, Henan, Jiangsu and Guangxi Province, China, between 2022 and 2023. Positive DNA/cDNA for ASFV, PRV, and PPV were obtained from the Exotic Disease Surveillance and Research Center of the China Animal Health and Epidemiology Center (CAHEC). Complementary DNA for porcine circovirus type 2 (PCV2), transmissible gastroenteritis virus (TGEV), porcine reproductive and respiratory syndrome virus (PRRSV), and classical swine fever virus (CSFV) were derived from archived specimens previously validated by PCR and RT-PCR. All swine pathogen-specific nucleic acids were maintained in our laboratory biorepository for targeted diagnostic applications.

### Primers and probes

Target-specific primers and hydrolysis probes were designed using Primer5.0 software for multiplex droplet digital PCR (ddPCR) detection, targeting the ASFV P72 gene, PRV gE gene, and PPV NS1 gene. These oligonucleotides were synthesized by Shanghai Bioengineering Co., Ltd. The ASFV probe incorporated FAM fluorophore, the PRV probe utilized CY5, and the PPV probe employed HEX for signal detection. Detailed primer and probe sequences are provided in Table 1.

**Table 1:** The primers and probes for detection of ASFV, PRV, and PPV.

Primer/Probe name	Sequence (5′–3′)	Target gene	Length (bp)	Source
ASFV-F	CCAGTAGACGCAATATACGCTTTA	P72	79	Designed in this study
ASFV-R	AGTTCATTATTCGTGAGCGAGAT			
ASFV-P	ACCATGGTTTATCCCAGGAGTCATTAATGA			
PRV-F	CTTCCACTCGCAGCTCTTC	gE	100	Designed in this study
PRV-R	CAGCGTGGCGGTAAAGT			
PRV-P	ACACGTTCGACCTGATGCCG			
PPV-F	GGAGGACCTGGATTTAGCTTTAG	NS1	118	Designed in this study
PPV-R	TGGACTTGGTGTCCGTATTG			
PPV-P	TGGAGCCGTGGAGCGAGCCAA			

### Preparation of recombinant plasmids

The target gene segments were amplified following established protocols (21). Conserved regions of the ASFV p72, PRV gE, and PPV NS1 genes were cloned into the pUC57 vector to generate recombinant plasmids (pUC57-ASFV, pUC57-PRV, and pUC57-PPV). These constructs were outsourced to Shanghai Bio-Technology Co., Ltd. (Shanghai, China) for Sanger sequencing verification. Plasmid concentrations were quantified using a NanoDrop spectrophotometer (Thermo Fisher Scientific), and copy numbers were calculated based on molecular weight and Avogadro’s constant. The plasmids were diluted to a standardized concentration of 1.0 × 10^9^ copies/μL and stored at −20 °C until experimental use.

### Multiplex ddPCR reaction conditions

All droplet digital PCR (ddPCR) assays were conducted using the Sniper DQ24 Pro^™^ ddPCR System, which integrates a droplet generator and automated fluorescence reader. The 25 μL triplex reaction mixture contained: 10 μL of 2 × ddPCR master mix, ASFV-specific primers (500 nM), FAM-labeled probe (200 nM), PRV-specific primers (500 nM), CY5-labeled probe (200 nM), PPV-specific primers (600 nM), HEX-labeled probe (320 nM), 0.5 μL droplet stabilizer, 1 μL Mg^2+^ solution, 1 μL template DNA, and 2.71 μL nuclease-free water were mixed to create a 25 μL triplex ddPCR reaction (Table 2). The PCR program was then started, with the following amplification program: 60 °C for 5 min, 95 °C for 20 s, 95 °C for 10 s for a total of 40 cycles, and then 58 °C for 30 s. Data analysis was performed using Sniper software.

**Table 2:** Multiplex ddPCR reaction system.

Reagents	Voluments (μL)	Final concentration (nM)
Taq Probe Master Mix	10	/
ddPCR Cy5.5 dyestuff	0.5	/
Mg^2+^	1	/
ASFV-F (10 μM)	1.25	500
ASFV-R (10 μM)	1.25	500
ASFV-P (10 μM)	0.5	200
PRV-F (10 μM)	1.25	500
PRV-R (10 μM)	1.25	500
PRV-P (10 μM)	0.5	200
PPV-F (10 μM)	1.5	600
PPV-R (10 μM)	1.5	600
PPV-P (10 μM)	0.8	320
Template	1	/
RNase free H_2_O	Up to 25	/

In the multiplex ddPCR workflow, each reaction generates 16,000–24,000 analyzable droplets. For robust data analysis, we established a minimum threshold of ≥18,000 valid droplets per reaction. The assay demonstrates linear quantification capability across a dynamic range of 0.2–20,000 copies/μL. Notably, the SightPro platform employs automated thresholding algorithms to precisely differentiate positive from negative droplets, enhancing detection specificity and minimizing subjective interpretation.

### Specificity evaluation

Assay specificity was validated using DNA/cDNA samples and recombinant plasmids from seven major swine pathogens: African swine fever virus (ASFV), pseudorabies virus (PRV), porcine parvovirus (PPV), porcine reproductive and respiratory syndrome virus (PRRSV), porcine circovirus type 2 (PCV2), transmissible gastroenteritis virus (TGEV), and classical swine fever virus (CSFV). All samples were analyzed in triplicate using the multiplex ddPCR platform to confirm cross-reactivity and target specificity.

### Sensitivity assessment

The limit of detection (LOD) was determined by serial dilution (10^5^–10^0^ copies/μL) of ASFV, PRV, and PPV positive DNA/plasmid standards in nuclease-free water. Three independent ddPCR experiments were performed at each dilution concentration to rigorously ensure the reliability and reproducibility of the experimental outcomes, adhering to standardized protocols for methodological robustness. False-negative rates were evaluated across the dilution series to establish the lowest detectable concentration with 95% confidence.

### Reproducibility testing

Intra- and inter-assay reproducibility were assessed using three plasmid concentrations (1 × 10^1^, 1 × 10^2^, and 1 × 10^3^ copies/μL) tested in triplicate within a single run (intra-assay) and across three independent batches (inter-assay). Coefficients of variation (CV) were calculated for each concentration level to quantify precision.

### Clinical sample testing

A total of 217 clinical samples stored at the Exotic Disease Surveillance and Research Center of the China Animal Health and Epidemiology Center (CAHEC) were analyzed using both the developed multiplex ddPCR and reference qPCR methods. Viral nucleic acids were extracted from all samples using the Tiangen Virus DNA Extraction Kit following standardized protocols. Each reaction included nuclease-free water as a negative control and quantified DNA from ASFV, PRV, and PPV as positive controls to ensure assay validity.

## Results

### Optimization of triplex ddPCR conditions

**Figure 1:**
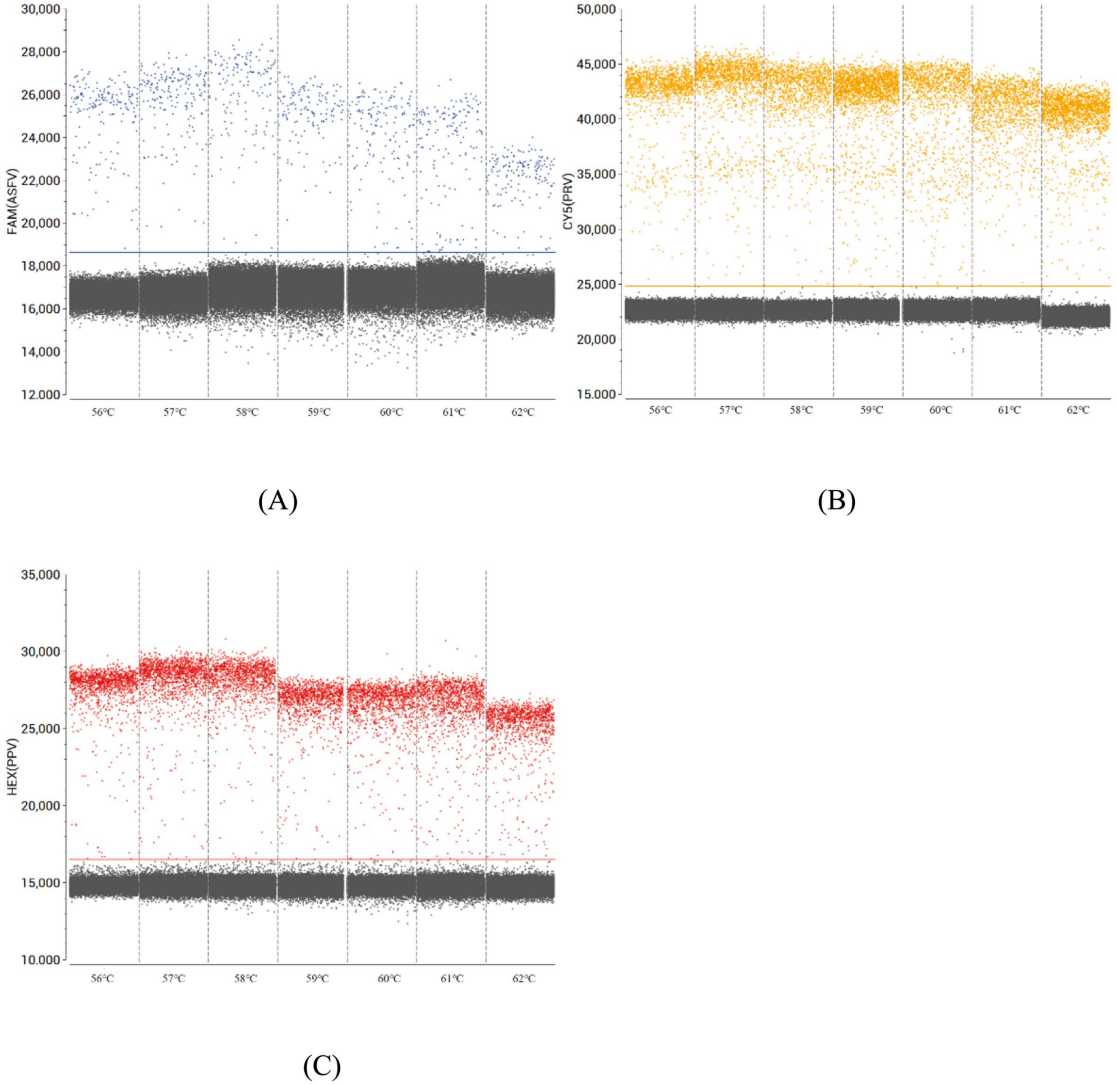
Optimization of annealing temperatures for multiplex ddPCR assays. The annealing temperature gradient for multiplex ddPCR assays targeting ASFV **(A)**, PRV **(B)**, and PPV **(C)** was evaluated across a range of 56 °C to 62 °C in 1 °C increments.

**Figure 2:**
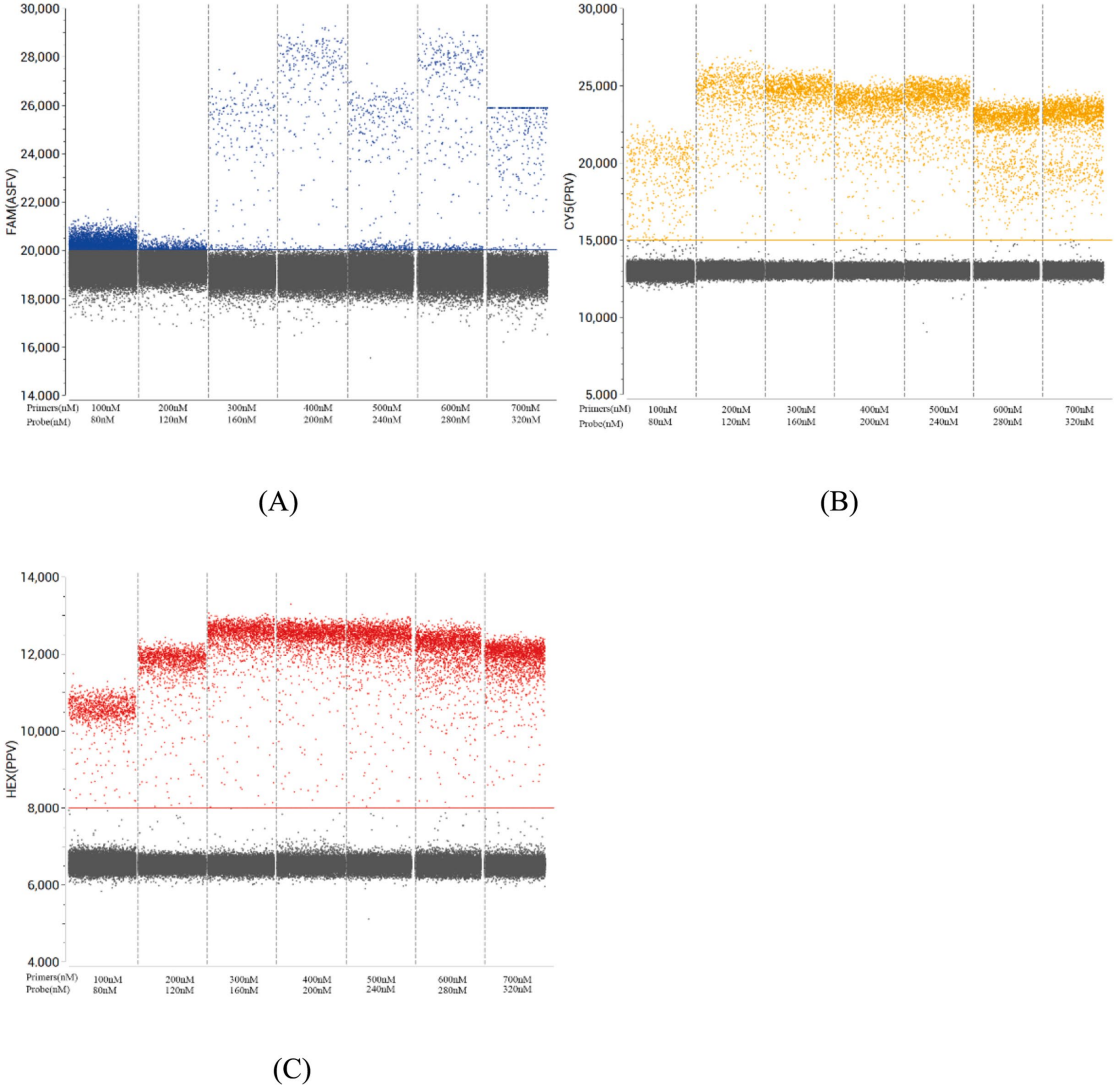
Optimization of primer and probe concentrations for multiplex ddPCR detection of ASFV **(A)**, PRV **(B)**, and PPV **(C)**. The fluorescence amplitudes of different combinations of primer and probe concentrations for the multiplex ddPCR.

Primer and probe concentrations alongside annealing temperatures were systematically optimized for triplex ddPCR using pUC57-ASFV, pUC57-PRV, and pUC57-PPV recombinant plasmids. A serial 10-fold dilution series (1.0 × 10^9^ to 1.0 × 10^0^ copies/μL) was prepared for each plasmid, with 1.0 × 10^3^ copies/μL selected as the working concentration for initial temperature screening. Since the concentration falls within the moderate range of the plasmid dilution series, it ensures adequate signal intensity for fluorescence detection while preventing signal saturation at higher concentrations, thereby enabling more accurate evaluation of amplification efficiency across different annealing temperatures. Subsequently, we evaluated annealing temperatures in the range of 56 °C to 62 °C, identifying 58 °C as optimal based on the maximal fluorescence amplitude observed in thermal gradient experiments (Figure 1). Subsequent optimization focused on primer-probe combinations at 58 °C using the 1.0 × 10^3^ copies/μL template. Concentration matrices were tested to achieve: (1) maximal fluorescence amplitude differential between target-positive (colored clusters) and negative droplets (gray clusters), and (2) sharp signal discrimination with minimal overlap. The final optimized concentrations (Table 2) were determined through iterative adjustment of primer and probe stoichiometry, validated by clear cluster separation in two-dimensional fluorescence plots (Figure 2).

After optimizing the conditions, we successfully developed a triplex ddPCR method. The reaction mixture, with a total volume of 25 μL, consisted of the following components: 10 μL Taq Probe Master Mix, 0.5 μL Cy5.5 dye, 1.25 μL each of ASFV primers (10 μM), 0.5 μL ASFV probe (10 μM), 1.25 μL each of PRV primers (10 μM), 0.5 μL PRV probe (10 μM), 1.5 μL each of PPV primers (10 μM), 0.8 μL PPV probe (10 μM), 1 μL DNA template, 0.5 μL Cy5.5 dye, 1 μL Mg^2+^ solution, and 2.7 μL RNase-free water (Table 3). The ddPCR amplification program was: 60 °C for 5 min, 95 °C for 20 s, and finally 40 cycles of 95 °C for 10 s and 58 °C for 30 s. After amplification, the absolute concentration of each sample is automatically reported using the Sniper system.

**Table 3:** Repeatability and reproducibility analysis of intragroup positive controls at different concentrations using multiplex digital PCR.

Standard plasmid	ASFV		PRV		PPV	
	Copy number ($\bar{x}$ ± *s*)	CV%	Copy number ($\bar{x}$ ± *s*)	CV%	Copy number ($\bar{x}$ ± *s*)	CV%
10^3^	1835.61 ± 22.96	1.25%	2651.52 ± 40.87	1.54%	2274.63 ± 21.61	0.95%
10^2^	169.53 ± 7.49	4.42%	321.43 ± 3.46	1.08%	244.38 ± 3.89	1.59%
10^1^	21.98 ± 0.83	3.77%	30.56 ± 1.05	3.44%	26.86 ± 0.90	3.35%

$\bar{x}$ is the mean value and *s* is the standard deviation.

### Triplex ddPCR specificity

**Figure 3:**
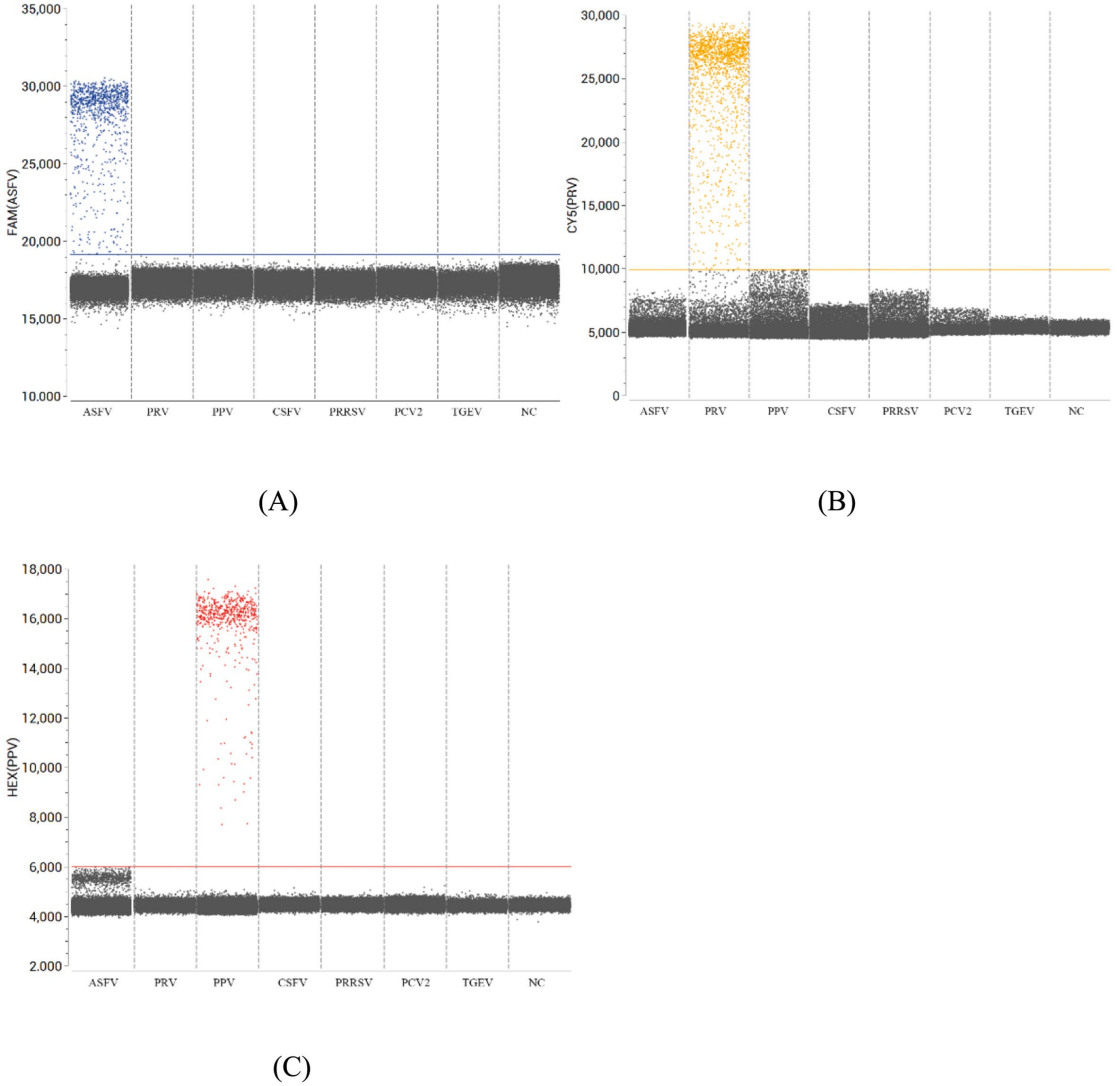
Specificity analysis of the multiplex ddPCR for detection of ASFV **(A)**, PRV **(B)**, and PPV **(C)**. The fluorescence amplitudes of ASFV, PRV, PPV, CSFV, PRRSV, PCV2, and TGEV were showed. NC, no template control.

The specificity of the triplex ddPCR assay was evaluated using standard plasmids (pUC57-ASFV, pUC57-PRV, pUC57-PPV) as positive controls, nucleic acids from prevalent swine viruses (CSFV, PRRSV, PCV2, TGEV) as test samples, and nuclease-free water as a negative control. Fluorescence signals were exclusively detected in the ASFV, PRV, and PPV channels, with no cross-reactivity observed in non-target viral samples or negative controls. This distinct signal separation (Figure 3) confirmed the assay’s high specificity for the three target pathogens.

### Repeatability of triplex ddPCR

For repeatability analysis, triplicate ddPCR reactions were performed using pUC57-ASFV, pUC57-PRV, and pUC57-PPV plasmids at concentrations of 1.0 × 10^3^, 1.0 × 10^2^, and 1.0 × 10^1^ copies/μL. To evaluate reproducibility, the same plasmid dilutions were tested in three independent experiments conducted on separate days. The intra-assay coefficient of variation (CV) ranged from 0.95 to 4.42%, while the inter-assay CV spanned 1.10 to 4.37%. These results confirm the triplex ddPCR assay’s high precision and robust reproducibility across different concentrations and experimental conditions (Tables 3, 4).

**Table 4:** Repeatability and reproducibility analysis of intergroup positive controls at different concentrations using multiplex digital PCR.

Standard plasmid	ASFV		PRV		PPV	
	Copy number ($\bar{x}$ ± *s*)	CV%	Copy number ($\bar{x}$ ± *s*)	CV%	Copy number ($\bar{x}$ ± *s*)	CV%
10^3^	2192.00 ± 23.25	1.10%	2331.88 ± 27.34	1.17%	2507.63 ± 42.86	1.71%
10^2^	198.30 ± 4.84	2.44%	232.04 ± 2.81	1.21%	203.86 ± 8.91	4.37%
10^1^	22.37 ± 0.77	3.46%	24.09 ± 0.49	2.03%	24.01 ± 0.82	3.42%

$\bar{x}$ is the mean value and *s* is the standard deviation.

### Comparison analysis of the sensitivity and standard curves between the triplex ddPCR and triplex qPCR

**Figure 4:**
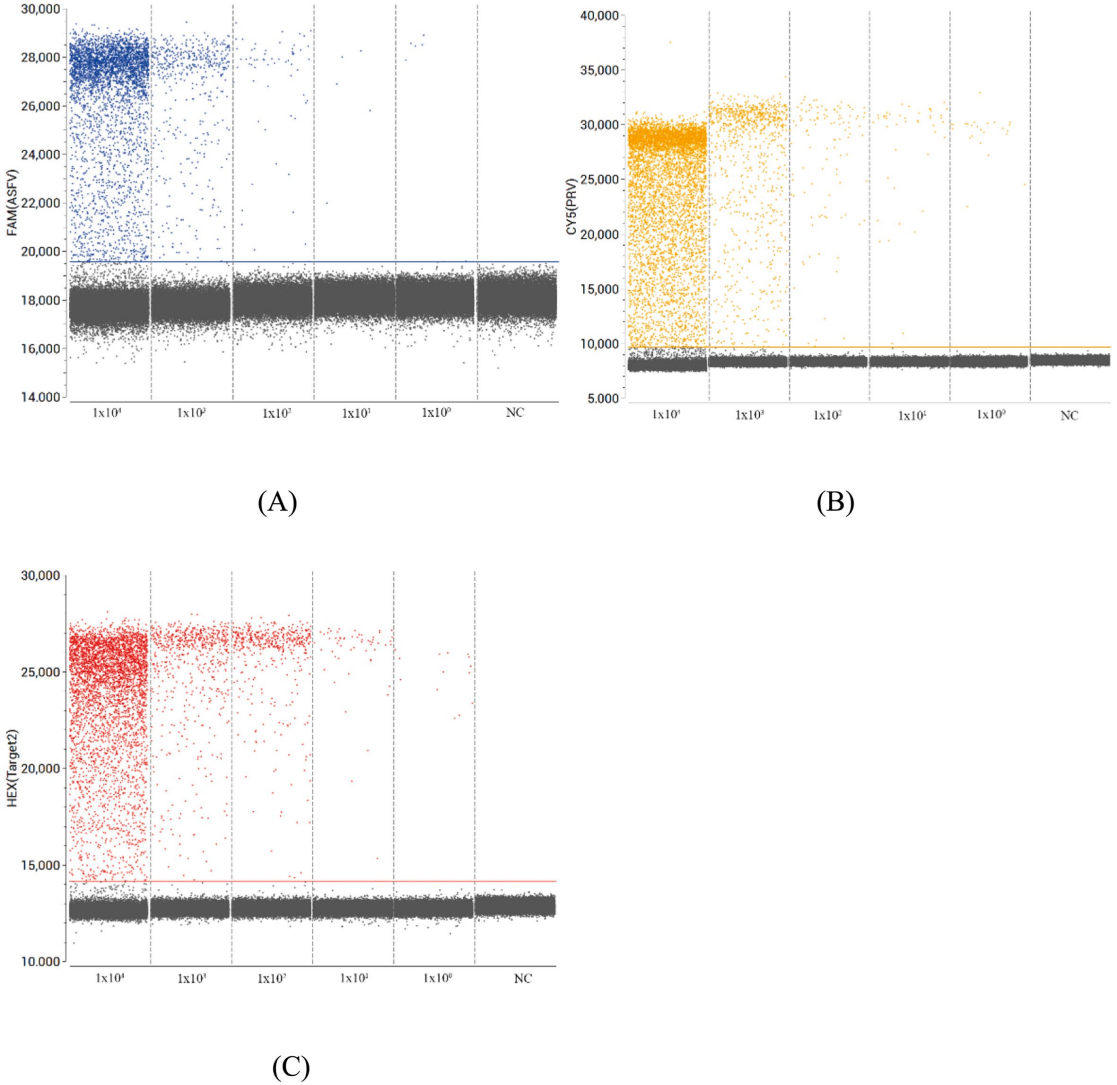
Performances of the triplex ddPCR assay by target DNA from 1 × 10^4^ to 1 × 10^0^ copies/μL. **(A)** Detection results of the target ASFV. **(B)** Detection results of the target PRV. **(C)** Detection results of the target PPV. NC, no template control.

**Figure 5:**
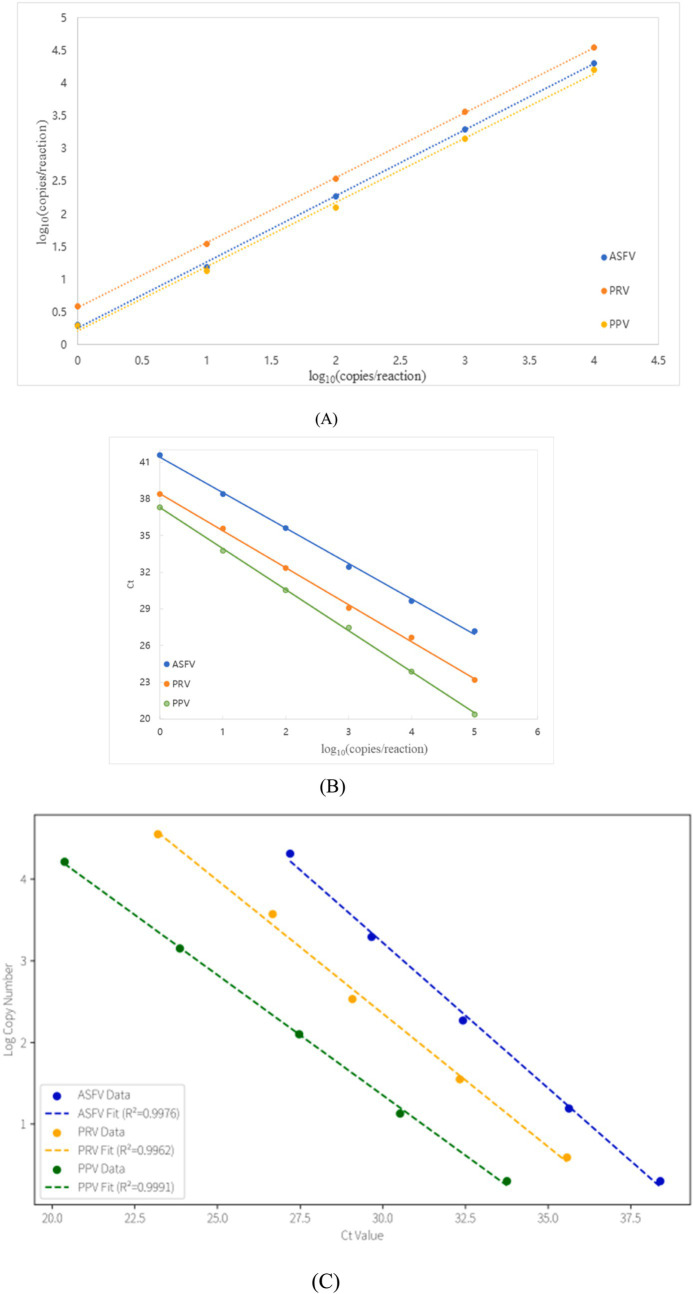
Standard curves of the multiplex ddPCR **(A)**, the multiplex qPCR **(B)**, and the correlation between these two methods **(C)**. The 10-fold serially diluted p-ASFV, p-PRV and p-PPV standard plasmids from 1 × 10^5^ to 1 × 10^0^ copies/μL were used to generate the standard curves. The correlation between these two methods was acquired by plotting the logarithm of absolute measured values of the multiplex ddPCR against the logarithm of cycle threshold (Ct) values of the multiplex qPCR.

Sensitivity was assessed using serial dilutions (1 × 10^4^ to 1 × 10^0^ copies/μL) of standard plasmids. The triplex ddPCR demonstrated limits of detection (LOD) at 0.08 copies/μL for ASFV, 3.41 copies/μL for PRV, and 3.38 copies/μL for PPV. Direct comparison with triplex qPCR using identical plasmid concentrations revealed qPCR’s LOD at 1 × 10^1^ copies/μL, confirming ddPCR’s 10-fold higher sensitivity (Figure 4). Standard curves generated from 1 × 10^5^ to 1 × 10^0^ copies/μL showed strong linearity for both methods: ddPCR yielded *R*^2^ values of 0.9992 (ASFV), 0.9998 (PRV), and 0.9977 (PPV), while qPCR produced *R*^2^ values of 0.9988 (ASFV), 0.9986 (PRV), and 0.9994 (PPV). Correlation coefficients between the two methods reached 0.9976 for ASFV, 0.9962 for PRV, and 0.9991 for PPV, indicating excellent agreement (Figure 5).

### Clinical performance of triplex ddPCR

The established triplex ddPCR and qPCR methods were applied to analyze 217 clinical samples. Using ddPCR, single infection rates were detected at 32.26% for ASFV, 4% for PRV, and 26.73% for PPV. Mixed infection rates included 21.66% for ASFV + PPV, 0.5% for ASFV + PRV, 0.92% for PPV + PRV, and 2.76% for triple co-infections. Comparative qPCR analysis showed lower detection rates: 26.27% (ASFV), 3.69% (PRV), 23.04% (PPV) for single infections, and 11.98% (ASFV + PPV), 0.46% (ASFV + PRV), 0.46% (PPV + PRV), and 0.92% (triple co-infections) for mixed infections. The triplex ddPCR demonstrated superior sensitivity and higher positive detection rates compared to qPCR, with concordance rates between the two methods reaching 94.01% (ASFV), 99.54% (PRV), and 96.31% (PPV) (Table 5). To further quantify the diagnostic consistency between triple ddPCR and qPCR, we calculated key metrics including the positive predictive value (PPV), negative predictive value (NPV), and Cohen’s kappa coefficient. In a cohort of 217 clinical samples, the overall PPV and NPV were determined to be 84.3 and 98.2%, respectively. The Cohen’s kappa coefficient was calculated as 0.85 [95% confidence interval (CI): 0.79–0.91], indicating almost perfect agreement between these two detection methods (Table 6). These findings confirm the enhanced diagnostic performance of ddPCR for multiplex pathogen detection in clinical settings.

**Table 5:** Multiplex ddPCR clinical results.

Detection method	Detection results (positive samples/total samples)		
	ASFV	PRV	PPV
ddPCR	70/217	9/217	58/217
qPCR	57/217	8/217	50/217
Coincidence rates	94.01%	99.54%	96.31%

**Table 6:** Consistency between multiplex ddPCR and multiplex qPCR.

Virus	Positive predictive value	Negative predictive value	Cohen’s kappa
ASFV	81.4% (57/70)	100% (147/147)	0.86
PRV	88.9% (8/9)	100% (208/208)	0.94
PPV	86.2% (50/58)	100% (159/159)	0.90
Overall consistency	84.3%	98.2%	0.85

## Discussion

The economic impact of sow reproductive failure remains a critical concern in swine production systems, with ASF virus (ASFV), pseudorabies virus (PRV), and porcine parvovirus (PPV) sharing overlapping clinical presentations that confound field diagnosis and control measures. While prior studies have reported multiplex qPCR assays for differential diagnosis of these pathogens (Hu et al., 2015; Shi et al., 2016; Wu et al., 2018; Zeng et al., 2014), and novel single-plex ddPCR methods for ASFV and PRV detection (Jia et al., 2021; Ren et al., 2018), no multiplex ddPCR platform has been previously described for simultaneous ASFV/PRV/PPV detection. This study establishes the first triplex droplet digital PCR (ddPCR) system capable of concurrently identifying these three economically significant pathogens.

Quantitative PCR (qPCR) remains the predominant detection method in veterinary diagnostics. King et al. (2003) developed a qPCR assay for rapid ASFV diagnosis, while Cheng et al. (2021) established a gB gene-targeted qPCR method for PRV detection in porcine saliva and nasal swabs. Gava et al. (2015) reported an ORF3 gene-based qPCR system for porcine parvovirus 4 (PPV4) quantification in field samples and epidemiological studies. Multiplex qPCR approaches have also been described, including Liu’s et al. (2023) triplex assay for simultaneous ASFV/PCV2/PRV detection and Pérez’s et al. (2012) quadruplex qPCR for rapid diagnosis of TTSuV1/PCV2/PRV/PPV in clinical practice.

Despite its widespread use, qPCR exhibits inherent limitations: dependency on standard curve calibration, susceptibility to PCR inhibitors, and reduced sensitivity for low-copy-number targets. Droplet digital PCR (ddPCR), a third-generation technology, effectively overcomes these constraints. By partitioning samples into nanoliter-scale droplets, ddPCR enables single-molecule amplification through Poisson distribution-based absolute quantification, eliminating the need for standard curves (Kuypers and Jerome, 2017; Quan et al., 2018). End-point fluorescence measurement, independent of Ct values, further minimizes inhibitor interference. Our comparative analysis demonstrated ddPCR’s superior performance: 10-fold greater sensitivity than qPCR, along with significantly lower inter-assay (1.2% vs. 5.8%) and intra-assay (0.8% vs. 3.2%) coefficients of variation. While both methods showed consistent results for moderate/high viral loads, ddPCR reliably detected low-concentration environmental samples and asymptomatic carriers where qPCR yielded false negatives. These findings confirm ddPCR’s enhanced reliability for low-viral-load detection, positioning it as a superior clinical tool for applications requiring absolute quantification or operation in inhibitor-rich matrices. Although digital PCR exhibits exceptional stability in samples with low viral loads, components in complex matrices such as blood and tissue fluid may cause uneven droplet generation, thereby increasing the risk of false negatives (Sidstedt et al., 2018). The detection costs associated with new detection methods may exceed those of traditional approaches. Digital PCR’s limited throughput and elevated per-sample costs present challenges for complete replacement of qPCR workflows, except in scenarios requiring high specificity/sensitivity applications where its superior performance justifies the additional expense (Luthra et al., 2016). In the realm of disease prevention and control, ddPCR, as a novel detection technology, has demonstrated substantial advantages; however, its translation from laboratory settings to field applications continues to confront multiple challenges. Future research should prioritize enhancing ddPCR throughput and advancing droplet generation/detection technologies to address the demands of large-scale epidemic surveillance, thereby optimizing its applicability in real-world disease control scenarios.

China reported its first African swine fever (ASF) outbreak in 2018, which rapidly spread across 31 provinces, causing severe economic losses to the swine industry (Zhou et al., 2018). The coexistence of wild-type, recombinant, and attenuated ASFV strains has significantly increased prevention and control challenges for pig farms. Epidemiological studies revealed a 57.14% ASFV positivity rate in Guangxi Province (2019–2020), indicating persistent viral circulation (Shi et al., 2022). PRV, a common swine pathogen, remains endemic in China: serum surveys showed 57.8% positivity in Shandong Province (2013–2016) (Gu et al., 2018), 30.14% in Henan Province (2018–2019) (Zheng et al., 2021), and 25.04% nationwide, with 55.96% positivity in free-range farms (Zhao et al., 2023). PPV, a later-identified but widespread pathogen in China, causes reproductive disorders and facilitates viral co-infections. Mixed infection rates include 5% for PPV/PCV2 in Zhejiang (Rao et al., 2014) and 4.8% for PPV/PCV2/PRRSV/CSFV in Guizhou (Zeng et al., 2014). Notably, ASFV co-infections cannot be excluded due to overlapping clinical presentations with CSFV and PRRSV. This study analyzed 217 clinical samples, revealing 32.26% ASFV positivity, 4% PRV, and 26.73% PPV. Mixed infections included ASFV + PRV (21.66%), ASFV + PPV (0.5%), PRV + PPV (0.92%), and ASFV + PRV + PPV (2.76%), underscoring the prevalence of multi-pathogen challenges. The developed triplex ddPCR platform offers a critical tool for efficient differential diagnosis, with superior sensitivity enabling accurate detection of ASFV, PRV, and PPV during early/low-viral-load stages. This capability is pivotal for rapid infection source identification, precise culling, and outbreak containment. Clinical application of the method demonstrated its value in detecting subclinical or persistent infections, thereby mitigating potential economic losses in swine production systems.

In summary, the multiplex ddPCR assay developed in this study demonstrates superior specificity and reproducibility compared to conventional diagnostic approaches, enabling precise identification and quantification of pathogens in complex samples. This platform provides unprecedented sensitivity for early detection of asymptomatic or low-viral-load infections, facilitating timely targeted interventions to prevent disease spread and mitigate associated economic losses. Notably, this represents the first reported multiplex ddPCR method for simultaneous ASFV, PRV, and PPV detection, offering a robust technical framework for veterinary surveillance programs. By addressing critical limitations of existing methods, our approach has direct applications in outbreak response, biosecurity management, and evidence-based control strategies for swine pathogens.

## Data Availability

The original contributions presented in the study are included in the article/Supplementary material, further inquiries can be directed to the corresponding authors.
