# Supplementary material for: Development and validation of a novel triplex droplet digital PCR assay for simultaneous detection of African swine fever virus, pseudorabies virus, and porcine parvovirus
Source: Front Microbiol. 2026 Jan 6;16:1710807. doi: 10.3389/fmicb.2025.1710807 (PMC12815706; doi:10.3389/fmicb.2025.1710807)
Supplement: Supplementary file 1 [file Table_1.DOCX]

Table1 Primers and probes utilized for the reference qPCR-based detection of ASFV, PRV, and PPV

| ASFV-F | CCCAGGRGATAAAATGACTG |  |  |  |
| --- | --- | --- | --- | --- |
| ASFV-R | CACTRGTTCCCTCCACCGATA | P72 | 113 | WOAH |
| ASFV-P | TCCTGGCCRACCAAGTGCTT |  |  |  |
| PRV-F | GCTCCTTCGTGATGACGTG |  |  |  |
| PRV-R | GTACACCGGAGAGAGCATGT | gE | 131 | (Liu et al., 2023) |
| PRV-P | CTGCGTGCTGTGCTCCCGGC |  |  |  |
| PPV-F | GAAGACTGGATGATGACATCCA |  |  |  |
| PPV-R | TGCTTTTTTTGTGTGTGTGTAGAGTAA | NS1 | 123 | (Song et al., 2010) |
| PPV-P | AATGATGGCTCAAACCGGAGGAGA |  |  |  |

Note: The letter "R" in the nucleotide sequence denotes a mixed base comprising both adenine (A) and guanine (G).

The initial concentrations of the primers were set at 0.6μL each for ASFV-p72 qF, ASFV-p72 qR, PRV gE qF, PRV gE qR, PPV-NS1F, and PPV-NS1R. The initial concentrations of the probes for ASFV, PRV, and PPV were uniformly 10μM, with a volume of 0.3μL used for each. The 2×qPCR master mix was added in a volume of 10μL, while the DNA template and ddH_2_O were incorporated at volumes of 2μL and 1.3μL, respectively, culminating in a total reaction volume of 25 microliters.

The qPCR reaction protocol proceeded as follows: an initial pre-denaturation step at 95°C for 3 minutes was followed by 40 cycles, each consisting of a 10-second denaturation at 95°C, a 10-second annealing at 58°C, and a 20-second extension at 72°C. Fluorescent channels 1-3 were employed to amplify three genes: ASFV p72 (labeled with FAM), PRV gE (labeled with Cy5), and PPV NS1 (labeled with HEX). Ultimately, all fluorescence signals were captured by real-time PCR instruments for analysis.

References

Liu, H., Zou, J., Liu, R., Chen, J., Li, X., Zheng, H., Li, L., Zhou, B., 2023. Development of a TaqMan-Probe-Based Multiplex Real-Time PCR for the Simultaneous Detection of African Swine Fever Virus, Porcine Circovirus 2, and Pseudorabies Virus in East China from 2020 to 2022. Veterinary Sciences, 106.

Song, C., Zhu, C., Zhang, C., Cui, S., 2010. Detection of porcine parvovirus using a taqman-based real-time pcr with primers and probe designed for the NS1 gene. Virology journal 7, 353.
